# Circumventing a challenging aspect of crystal structure determination from powder diffraction data

**DOI:** 10.1107/S2052520622003717

**Published:** 2022-04-01

**Authors:** Kenneth D. M. Harris

**Affiliations:** a School of Chemistry, Cardiff University, Park Place, Cardiff, CF10 3AT, United Kingdom

**Keywords:** crystal structure determination, powder diffraction data, indexing, unit-cell determination, global optimization

## Abstract

Schmidt and co-workers [ *Acta Cryst.* (2022), B**78**, 195–213], report a strategy for structure determination from powder XRD data in which unit-cell determination and structure solution are combined within a single process, rather than handling them as sequential stages on the structure determination pathway. This strategy offers the prospect to achieve successful structure determination in cases for which conventional approaches for indexing powder XRD data prove to be challenging.

Although recent decades have seen significant progress in the opportunities for carrying out crystal structure determination directly from powder X-ray diffraction (XRD) data (McCusker, 1991 ▸; Harris *et al.*, 2001 ▸; David & Shankland, 2008 ▸; Etter & Dinnebier, 2014 ▸; Černý, 2017 ▸), this task is still considerably less routine than structure determination from single-crystal XRD data. In some cases, the structure determination process fails at the first stage, namely unit-cell determination (often referred to as ‘indexing’ the powder XRD data), such that the process cannot progress to the subsequent structure solution and structure refinement stages. For reasons highlighted below, unit-cell determination from powder XRD data can encounter challenges under certain circumstances, which may represent an insurmountable hurdle in the structure determination process. Specific challenges arise in cases of poor-quality powder XRD data (for example, when severe peak broadening arises due to poor crystallinity of the sample) and/or poor-quality samples (for example, containing an unknown impurity crystalline phase).

In light of the challenges that can arise in unit-cell determination, the report of a strategy by Schmidt and co-workers (Habermehl *et al.*, 2022 ▸) that essentially circumvents the indexing step in structure determination from powder XRD data will be welcomed with interest by practitioners in the field. However, to place this development in context, it is relevant first to explain briefly the methodology for indexing powder XRD data, and the reasons that challenges may be encountered in this stage of the structure determination process.

Unit-cell determination involves analysis of the ‘positions’ of Bragg reflections in the powder XRD data, where ‘position’ refers to the diffraction angle 2θ or the corresponding *d*-spacing. The aim is to find a set of unit-cell parameters (*a*, *b*, *c*, α, β, γ), together with the corresponding set of Miller indices (*h*, *k*, *l*) for each Bragg reflection, that successfully account for the positions of all peaks in the powder XRD data. Typically, the positions of about 20 peaks are used in the indexing process, measured from the low-2θ region of the experimental powder XRD data, where the extent of peak overlap is less than in the high-2θ region. It is important to note that the aim of the indexing stage is to establish a good initial approximation to the correct unit cell, which is then improved in subsequent profile-fitting calculations [typically carried out using the algorithms of Pawley (1981 ▸) or Le Bail (1988 ▸)] to generate a more accurate set of unit-cell parameters for use in subsequent structure solution and refinement calculations.

In the early days, when powder XRD data were measured on photographic film and data analysis relied on mathematical insight rather than computational power, the challenges associated with unit-cell determination were already well recognized (Henry *et al.*, 1961 ▸), and a number of approaches were pioneered for indexing such data (Hesse, 1948 ▸; Vand, 1948 ▸; Lipson, 1949 ▸; Ito, 1949 ▸; de Wolff, 1957 ▸). Subsequently, the emergence of computer technology stimulated the development of a wide range of new strategies for unit-cell determination from powder XRD data (Taupin, 1968 ▸; Visser, 1969 ▸; Kohlbeck & Hörl, 1976 ▸; Werner *et al.*, 1985 ▸; Boultif & Louër, 1991 ▸; Paszkowicz, 1996 ▸; Kariuki *et al.*, 1999 ▸; Coelho, 2003 ▸; Neumann, 2003 ▸; Habershon *et al.*, 2004 ▸; Le Bail, 2004 ▸; Altomare *et al.*, 2009 ▸; Oishi-Tomiyasu, 2014 ▸; Louër & Boultif, 2014 ▸), many of which form the basis of indexing programs that are widely used today.

With this large array of computational approaches at our disposal, unit-cell determination from powder XRD data often proceeds straightforwardly and successfully. However, a number of factors can limit the chances of success in specific circumstances. Two particularly serious situations are: (i) severe peak overlap in the powder XRD data (which may limit the ability to extract accurate values of peak positions, as required for indexing calculations, and can even obscure the existence of certain peaks that may be crucial for successful indexing), and (ii) the presence of a crystalline impurity phase in the powder sample (such that the set of peak positions used for attempted unit-cell determination actually arise from two different unit cells).

Problems of severe peak overlap arise in the case of samples that give broad peaks in the powder XRD pattern, for example due to poor crystallinity and/or particle size effects. In such cases, it may be challenging to extract a sufficient number of reliable peak positions from the powder XRD data to allow successful indexing. Furthermore, even for samples of good crystallinity, significant peak overlap can still arise in cases with large unit cells and/or low symmetry due to a high density of peaks in the powder XRD pattern, even though the individual peaks are relatively narrow. This situation often arises for organic molecular materials. In such cases, measuring the powder XRD data using a synchrotron radiation source may help to alleviate the extent of peak overlap by minimizing the instrumental contribution to peak widths, and hence may be conducive to successful indexing.

To assess whether failure to index a powder XRD pattern is due to the presence of an impurity crystalline phase in the powder sample, independent evidence may be established by other experimental techniques (particularly solid-state NMR) or simply by checking whether the powder XRD pattern contains peaks due to plausible impurity phases of known crystal structure (such as known polymorphs of the material of interest). In favourable cases, the impurity phase may be identified as a material of known structure, in which case the peaks due to the impurity phase can be readily excluded from the indexing calculation, allowing successful unit-cell determination using only the peaks for the phase of interest (for example: Al Rahal *et al.*, 2019 ▸). In some cases, the existence of two crystalline phases in a powder sample may be identified by observing that the powder XRD data contain two sets of peaks with significantly different peak widths (for example: Dinnebier *et al.*, 1997 ▸).

Of course, when attempts to index a powder XRD pattern prove to be problematic, other experimental techniques may be exploited to determine the unit-cell parameters for the material of interest, such as electron diffraction (for example: Gorelik *et al.*, 2009 ▸; Smalley *et al.*, 2022 ▸) or measurement of XRD data for magnetically oriented micro-crystalline arrays (Kimura & Kimura, 2018 ▸). Another approach to generate possible unit cells independently of the powder XRD data is to take advantage of crystal structure prediction algorithms (Price, 2014 ▸; Woodley *et al.*, 2020 ▸), which generate a set of energetically feasible crystal structures for the molecule of interest; in principle, one of the crystal structures may be found to give an acceptable match to an unindexed experimental powder XRD pattern (for example: Paulus *et al.*, 2007 ▸).

Although the approaches discussed above provide a wide range of opportunities to overcome problems encountered in indexing powder XRD data, the development of structure determination protocols that effectively circumvent the indexing stage may be seen as an attractive alternative. The strategy reported by Schmidt and co-workers (Habermehl *et al.*, 2022 ▸), entitled ‘*Structure determination from unindexed powder data from scratch by a global optimization approach using pattern comparison based on cross-correlation functions*’, achieves this objective by combining unit-cell determination and structure solution within a single process, rather than handling them as sequential stages on the structure determination pathway. The methodology, which is embodied within the program *FIDEL-GO*, requires only an experimental powder XRD pattern and knowledge of the molecular structure as input information. The strategy starts from a set of trial crystal structures generated by random selection of both the unit-cell parameters and the arrangement of molecules within the asymmetric unit, with the space groups of the trial structures selected from a set of ‘common’ space groups. The strategy then applies a multi-step procedure for global optimization, with the ultimate aim of generating structural models that give an acceptable quality of fit to the experimental powder XRD data by optimization of *both* the unit-cell parameters *and* the set of structural variables that define the atomic positions within the asymmetric unit. In common with standard practice in direct-space structure solution of molecular materials from powder XRD data (Harris *et al.*, 2001 ▸; David & Shankland, 2008 ▸), the structural models used in *FIDEL-GO* are typically based on the assumption of standard bond lengths and bond angles (although one example discussed by Schmidt and co-workers includes relaxation of certain bond lengths), and the set of structural variables comprises, for each crystallographically independent molecule, the position of the molecule in the unit cell, the orientation of the molecule with respect to the unit-cell axes and a set of variable torsion angles that define the molecular conformation.

Importantly, a critical aspect underlying the *FIDEL-GO* approach is the selection of an appropriate method to assess the quality of agreement between the powder XRD pattern calculated for each trial structural model and the experimental powder XRD pattern, as the similarity measure used for this assessment must be able to give a meaningful indication of the quality of agreement even when the unit cell for the trial structural model deviates significantly from the correct unit cell. This task is achieved using a similarity measure based on a weighted cross-correlation function, specifically the generalized similarity measure *S*_12_ defined previously by de Gelder *et al.* (2001 ▸).

Following the optimization process, promising structural models generated by *FIDEL-GO* are subjected to an automated Rietveld fitting procedure to provide a more reliable assessment of the quality of fit to the experimental powder XRD data. The authors recommend that this stage is then followed by manual Rietveld refinement calculations as the final step of the process, to allow a robust ranking of the best candidate structures to be determined.

The authors note that their approach has a tendency to generate several different structures that give a similar quality of fit to the experimental powder XRD data. Clearly, under such circumstances, it is essential to further scrutinize the validity of the candidate structures using information from other computational (for example, DFT-D) and experimental (for example, solid-state NMR) techniques, in order to provide robust and conclusive evidence that the correct structure has actually been assigned on the basis of the final Rietveld refinement calculations. The application of methods for structure determination from PDF data (Billinge, 2019 ▸) for the same material may also be valuable in this regard (for example: Schlesinger *et al.*, 2021 ▸).

It is important to note that the concept of carrying out unit-cell determination and structure solution from powder XRD data synchronously within the same protocol has been reported in previous studies (Hofmann & Kuleshova, 2006 ▸; de Gelder *et al.*, 2008 ▸; Padgett *et al.*, 2007 ▸; Rapallo, 2009 ▸; Guguta *et al.*, 2019 ▸), which are based on a variety of different strategies for carrying out optimization of the fit to the experimental powder XRD data, in some cases incorporating information from calculations of the energetic properties of trial crystal structures. The workflow of the *FIDEL-GO* algorithm also includes the opportunity to carry out DFT-D calculations, particularly in the context of validation of promising candidate structures in the final stages of the structure determination process.

The successful application of the *FIDEL-GO* strategy is demonstrated by structure determination of four materials from powder XRD data of poor quality, described by the authors as ‘un-indexable’, typically containing only a relatively small number broad peaks. In comparison to current capabilities in the application of direct-space structure solution using high-quality (‘indexable’) powder XRD data, the selected materials represent structural problems of relatively low complexity, defined by a small number of structural variables (two of the four examples are rigid molecules, for which no variable torsion angles are required to define the intramolecular geometry, while the other two examples involve only a few variables to define the intramolecular geometry). Nevertheless, the successful structure determinations reported by Schmidt and co-workers clearly represent promising progress in demonstrating the feasibility of the *FIDEL-GO* strategy, and the success is particularly notable given the poor quality of the experimental powder XRD data used in some of their applications. Certainly, the results of future applications of *FIDEL-GO* to tackle structural problems of greater complexity, defined by a higher number of structural variables, will be eagerly awaited.

By demonstrating the prospect of achieving successful crystal structure determination from poor-quality powder XRD data, the *FIDEL-GO* strategy certainly offers a promising opportunity that may prove to be particularly beneficial when challenges are encountered in applying the conventional approaches for indexing powder XRD data.
